# Parental Incarceration, Child Adversity, and Child Health: A Strategic Comparison Approach

**DOI:** 10.3390/ijerph18073384

**Published:** 2021-03-25

**Authors:** Dylan B. Jackson, Alexander Testa, Daniel C. Semenza, Michael G. Vaughn

**Affiliations:** 1 Department of Population, Family, and Reproductive Health, Johns Hopkins Bloomberg School of Public Health, Baltimore, MD 21205, USA; 2 Department of Criminology & Criminal Justice, University of Texas at San Antonio, San Antonio, TX 78207, USA; alexander.testa@utsa.edu; 3 Department of Sociology, Anthropology & Criminal Justice, Rutgers University-Camden, Camden, NJ 08102, USA; daniel.semenza@rutgers.edu; 4 College for Public Health and Social Justice, Saint Louis University, Saint Louis, MO 63103, USA; michael.vaughn@slu.edu

**Keywords:** adverse childhood experiences, parental incarceration, health, children, stress, adversity, strategic comparison

## Abstract

*Background:* Research points to parental incarceration as an important social determinant of child health. Even so, studies examining the health impact of parental incarceration in the context of diverse childhood stressors and adversities are lacking. *Methods:* The present study uses a large, nationally representative sample to compare U.S. children who were exposed to parental incarceration to a strategic comparison group of U.S. children who were not exposed to parental incarceration, but were nonetheless exposed to alternative family stressors and adversities. *Results:* The initial findings generally reveal worse health among children exposed to parental incarceration compared to those who are not exposed. Even so, these differences were partially or completely attenuated when comparing the incarceration-exposed group to more comparable groups of children exposed to a varying degree of alternative stressors/adversities. *Conclusions:* Programmatic efforts targeting parental incarceration as a means of promoting child health may be enhanced by adequately addressing co-occurring family stressors and child adversities.

## 1. Introduction

Starting in the early 1970s, the United States began an unprecedented trajectory marked by the rapid increase in the use of incarceration. In what has become described as an era of mass incarceration [1], over the next four decades, the incarceration rate in the U.S. increased by 500%, resulting in approximately 2.2 million individuals currently behind bars [2,3]. A substantial amount of research has focused explicitly on understanding the consequences of incarceration for health and well-being [4,5,6]. Considering that approximately 2.6 million children have experienced the incarceration of a parent [7], there has also been a growing focus on understanding how this form of adversity can impact the health, well-being, and development of children [8,9]. Even so, most research to date investigates the adverse consequences of parental incarceration for health at later stages of life (i.e., young adulthood) [10,11,12,13], while fewer studies have detailed how parental incarceration can adversely impact health and well-being during childhood and adolescence [14,15,16].

Living in a household in which parents have been or are incarcerated is theorized to harm the health and well-being of children and adolescents because of the stress caused by this experience. Stress process theory posits that living in disadvantaged social contexts leads to differential exposure to social stressors that carry negative repercussions for health [17]. Incarceration is a well-documented social stressor that impacts the lives of individuals both during the period of incarceration, as well as in the short and long term following release from a correctional facility [15,18,19,20]. Indeed, during incarceration, individuals face a host of stressors including a lack of autonomy and privacy, exposure to violence, and being disconnected from friends and family [21,22,23]. Having a family member incarcerated is also a major stressor on families [24], a process so stressful it has been described as “doing time together” [21]. Post-release stressors continue to accumulate as a formerly incarcerated individual must re-establish ties with friends, family, the labor force, and civic society, all while facing a host of formal and informal barriers [25,26,27,28,29]. Furthermore, when the person experiencing incarceration is a parent, the stress may proliferate intergenerationally, such that the stressors experienced by a parent can have cascading consequences that negatively impact the well-being and development of children and adolescents [15,30].

Nonetheless, an ongoing challenge among extant research has been to isolate the impact of incarceration as a social stressor on the well-being of children and adolescents, considering those who spend time behind bars are typically exposed to a host of other social stressors aside from incarceration that can generate harm for children and adolescents [31,32]. Thus, three fundamental facts—(1) incarceration is a social stressor, (2) social stressors harm health, and (3) incarceration-exposed populations experience a multitude of social stressors—have led to two competing perspectives regarding the impact of parental incarceration for child and adolescent well-being: the unique effects and the selection effects perspective [31,33].

The unique effects perspective posits that a parent’s incarceration will independently and directly negatively impact the well-being of their children. As Shlafer and colleagues [33] explain, “parental incarceration is thought to pose some unique risk in a child’s life, in part as the result of parent-child separation, changes in living arrangements and caregivers, and other forms of disruption resulting from parental incarceration.” In contrast, the selection effects perspective surmises that other adverse childhood experiences and disadvantage that are commonly connected to incarceration lead to deleterious childhood outcomes, rather than parental incarceration itself. To be sure, the incarcerated population is not a representative segment of the general population, but rather the carceral population is disproportionately drawn from those with a multitude of competing adversities and stressful life events, including high rates of family instability, exposure to violence, substance use, mental health issues, and economic hardship [3,32,34]. Consequently, it may be exposure to these other childhood adversities and stressful life events—rather than exposure to parental incarceration—that interferes with child health and development. In other words, “children may be exposed to parental criminal behavior, or their parents may be less skilled as caregivers as a result of mental health or substance use issues. Thus, even when the parent is not incarcerated, these children may be at exceptional risk because of the contexts in which they are developing” [33].

In short, the unique effects and selection effects perspective propose two separate hypotheses. The unique effects perspective anticipates that net of confounding characteristics, incarceration should have a negative association with health and developmental outcomes, because incarceration is seen as a unique and potent social stressor [31,35,36]. In contrast, the selection effects perspective anticipates that risk factors associated with parental incarceration, rather than the incarceration itself, are responsible for any adverse childhood outcomes. The ongoing challenge, however, lies in formulating an analysis that can disentangle these two perspectives. To date, the most common approach is to control for observable characteristics related to demographics, socioeconomic status, and a variety of risk markers. However, most research in this vein is limited in two respects. First, the survey data that are typically employed are unable to account for a host of unobservable characteristics related to competing social stressors that may confound the association between parental incarceration and child well-being [37,38]. Second, differences between the incarcerated and non-incarcerated populations across a variety of characteristics are quite large [34], and standard regression techniques are often only able to adjust for small between-group differences [39]. This raises a key question of whether analyzing children who experience parental incarceration in reference to those who do not constitutes a proper comparison, and whether any differences found may result from differences in broader family stressors and child adversities [37]. In other words, it remains “unclear whether the difficulties that have been observed among children whose parents are incarcerated are due to the incarceration itself or to other adversities that children may have experienced” [31].

To account for this issue, recent studies have implemented strategic comparison groups using individuals who are similarly exposed to stressors, but differentially exposed to incarceration, as a control group [19,36,38,40,41]. Such an approach is beneficial since comparing those who have experienced similar, albeit different, social stressors can help account for some of the unobservable characteristics that may lead to these stressors. As Porter [19] notes, “using an appropriate comparison group can thus help account for (although not completely eliminate) unobserved heterogeneity.” For instance, a few studies have compared formerly incarcerated individuals to those convicted of a crime, but not incarcerated [19,40]. In disentangling the effects of parental incarceration on criminal offending of their children, Porter and King [38] compared children who experienced parental incarceration to those who had not experienced it but would in the future. The findings demonstrate that the implementation of a more comparable reference group substantially reduces the effect of parental incarceration on children’s delinquency. Another study by Testa and colleagues [36] employed a stressor-informed strategic comparison method that compared incarceration-exposed pregnant women to those who were not exposed to incarceration, but experienced differential exposure to stressors. Findings revealed that the adverse impact of incarceration exposure on maternal and infant health was substantially reduced after employing the stressor-informed comparison groups. Finally, a recent study by McCauley [41] examined risky sexual behaviors among youth exposed to household members incarceration by estimating associations in comparison to youth who experienced other forms of stress in the absence of incarceration. Findings indicated that linkages between household member incarceration and risky sexual behavior among youth were attributable in part to the co-occurring stressors linked to incarceration exposure.

The current study aims to extend prior research in two key ways. First, we examine the influence of incarceration on a variety of health and developmental markers in childhood and adolescence that have not been simultaneously investigated in previous research. To do so, we draw from contemporary and nationally representative data from the National Survey of Children’s Health (NSCH). Second, we expand upon previous research in this area by using reports of other forms of adverse childhood experiences to implement a stressor-informed strategic comparison group design that compares children with incarcerated parents to those who did not experience parental incarceration, but experience various social stressors that would be theorized to influence health and developmental outcomes under the stress process model [17,30]. In particular, the aim of this approach is to account for unobservable characteristics that may be associated with experiencing stressful events, while generating a more appropriate comparison group that allows us to test the hypothesis that incarceration is a unique social stressor that adversely impacts health and development for children and adolescents.

## 2. Materials and Methods

Data from the three most recent available cohorts (2016–2018) of the National Survey of Children’s Health (NSCH) are utilized in the current study. The NSCH is a survey of a cross-sectional weighted probability sample of U.S. children, ranging in age from 0 to 17 years. The survey is funded by HRSA’s Maternal and Child Health Bureau and conducted by the U.S. Census Bureau. Although a previous version of the NSCH was conducted three times between 2003 and 2012, a revised mail and web-based survey has been conducted each year since 2016, with 2018 being the most recent year available. The sample was taken from the Census Bureau’s Master Address File, which contains a complete listing of all known residences in the U.S. and the District of Columbia, and includes an administrative flag to identify households that are most likely to have children [42]. The survey assessed multiple, intersecting components of children’s lives and includes items that ask primary caregivers about the health of focal children across a variety of domains (e.g., daily health challenges, chronic physical conditions, developmental disorders, and mental health conditions) as well as their exposure to multiple forms of adversity (including parental incarceration). Additionally, the large, nationally representative sample (*N* = 102,341) pooled from years 2016, 2017, and 2018 facilitates the analysis of the association between fairly rare health conditions and forms of childhood adversity that would otherwise be impossible or challenging to capture with smaller local or regional samples. Given these features of the data, they are well suited to the present inquiry.

Importantly, 102,341 questionnaires were completed from 2016 to 2018 for all focal children ages 0 to 17 (2016 *N* = 50,212; 2017 *N* = 21,599; 2018 *N* = 30,530). To address missing observations in multivariate analyses, we present multiply imputed results calculated in STATA 15.1 (STATA Corp, College Station, TX, USA) using the MI commands (chained equations; 20 imputations). Results employing listwise deletion, however, were substantively identical, and results were invariant to the number of imputations employed (e.g., 5, 10).

### 2.1. Child Health Dimensions

In the present study, we follow the lead of Jackson, Posick, & Vaughn [43] and examine four distinct child health dimensions: health difficulties, chronic physical conditions, developmental disorders, and mental health conditions.

#### 2.1.1. Health Difficulties

First, we assessed health difficulties using parent/caregiver responses to seven items asking, whether during the 12 months prior to the survey, their child had had difficulty with or had experienced any of the following: breathing or other respiratory problems (such as wheezing or shortness of breath), eating or swallowing, digesting food (e.g., stomach/intestinal problems, constipation, or diarrhea), repeated or chronic physical pain (e.g., body pain, back pain), frequent or severe headaches (including migraines), toothaches, and bleeding gums. For each of these 7 health difficulties, children were assigned a value of 1 if their parent responded that they had experienced the health difficulty during the past 12 months, and a value of 0 if their parent responded that they had not experienced the health difficulty in the past 12 months. We aggregated these items into a count measure assessing the total number of health difficulties experienced by a given child. Subsequently, we also identified children who experience none of the health difficulties (78.3%), only one of the health difficulties (15.1%), and multiple health difficulties (6.6%), as a means of detecting differences in parental incarceration effects based on the clustering of health difficulties.

#### 2.1.2. Chronic Physical Conditions

Second, we assessed chronic physical conditions using parent/caregiver responses to six items asking about the following conditions: allergies, asthma, blood disorders, diabetes, heart conditions, and arthritis. In each case, parents were asked whether (1) a doctor or health care provider had ever told the adult caregiver/parent that the child had the condition and (2) whether the child currently had the condition. Children of caregivers who responded in the affirmative to both these questions were coded as having the condition (1), whereas all other children were coded as not having the condition (0). We aggregated these items into a count measure assessing the total number of chronic physical conditions experienced by a given child. Subsequently, we also identified children who experience none of the chronic physical conditions (73.6%), only one of the chronic physical conditions (20.9%), and multiple chronic physical conditions (5.5%), as a means of detecting differences in parental incarceration effects based on the clustering of chronic physical conditions.

#### 2.1.3. Developmental Disorders

Third, we assessed developmental disorders using parent/caregiver responses to six items asking about the following conditions: Tourette’s syndrome, intellectual disability, learning disability, speech disorder, autism, and developmental delay. In each case, parents were asked whether (1) a doctor or health care provider had ever told the adult caregiver/parent that the child had the condition and (2) whether the child currently had the condition. Children of caregivers who responded in the affirmative to both these questions were coded as having the condition (1), whereas all other children were coded as not having the condition (0). We summed these items into a count measure assessing the total number of developmental disorders experienced by a given child. Subsequently, we also identified children who experience none of the developmental disorders (89.6%), only one of the developmental disorders (5.6%), and multiple developmental disorders (4.8%), as a means of detecting differences in parental incarceration effects based on the clustering of developmental disorders.

#### 2.1.4. Mental Health Conditions

Finally, we assessed mental health conditions using parent/caregiver responses to four items asking about the following conditions: depression, anxiety, conduct problems, and ADHD. In each case, parents were asked whether (1) a doctor or health care provider had ever told the adult caregiver/parent that the child had the condition and (2) whether the child currently had the condition. In the case of conduct problems, parents were asked whether “a doctor, other health care provider, or educator” evert told them that their child had conduct problems. Teachers and school nurses were provided as examples of educators. Children of caregivers who responded in the affirmative to both these questions were coded as having the condition (1), whereas all other children were coded as not having the condition (0). We totaled these items into a count measure assessing the total number of mental health conditions experienced by a given child. Subsequently, we also identified children who experience none of the mental health conditions (84.2%), only one of the mental health conditions (8.3%), and multiple mental health conditions (7.5%), as a means of detecting differences in parental incarceration effects based on the clustering of mental health conditions.

#### 2.1.5. Health Conditions across Dimensions

To best illustrate the overall pattern of results, we also created a measure reflecting the extent to which children manifested health conditions across these four dimensions (i.e., health difficulties, chronic physical conditions, developmental disorders, and mental health conditions). To create this measure, we followed the lead of Jackson, Posick, and Vaughn [43] and summed binary measures of the presence/absence of any health condition within a given dimension across all four dimensions. Subsequently, we examined the association between parental incarceration and the number of health dimensions in which the focal children exhibited conditions/disorders.

### 2.2. Incarceration

In addition to being asked questions pertaining to children’s health, primary caregivers were also asked whether, to the best of their knowledge, the child had ever experienced a parent or guardian serving time in jail. Afterwards, primary caregivers were given the option to indicate *Yes* (1) or *No* (2). Response options were recoded so that children whose caregivers reported that they had experienced parental incarceration were assigned a value of 1 (6.4%), with all other children being assigned a value of 0.

### 2.3. Incarceration Strategic Comparisons

In addition to being asked questions pertaining to parent incarceration, primary caregivers were also asked to report on whether focal children had experienced eight other adverse childhood experiences during their lifetime [44], including parental divorce/separation, parent/guardian death, witnessing domestic violence in the home, witnessing/being the victim of neighborhood violence, living with anyone with a drug or alcohol problem, living with anyone with a mental illness or who was suicidal, discrimination, and extreme economic/material hardship. In a fashion similar to prior research [36,41], we employed data on these stressful, adverse experiences to create strategic comparison groups of children who had not experienced parent incarceration, but had nonetheless experienced other stressors/adversities to varying degrees. In order to create strategic comparison groups, children who had not experienced parental incarceration were divided into three groupings: (1) No Incarceration, No Adversity, (2) No Incarceration, Single Adversity, and (3) No Incarceration, Multiple Adversities.

#### 2.3.1. No Incarceration, No Adversity

Children were assigned a value of 1 if they had neither experienced parental incarceration nor any of the eight other forms of adversity assessed in the NSCH (59.6%). All other children were assigned a value of 0 on this measure.

#### 2.3.2. No Incarceration, Single Adversity

Children were assigned a value of 1 if they had not experienced parental incarceration, but had experienced one (and only one) of the other forms of adversity assessed in the NSCH (21.4%). All other children were assigned a value of 0 on this measure.

#### 2.3.3. No Incarceration, Multiple Adversities

Children were assigned a value of 1 if they had not experienced parental incarceration, but had experienced two or more of the other forms of adversity assessed in the NSCH (12.6%). All other children were assigned a value of 0 on this measure.

### 2.4. Covariates

The following covariates were also included in all multivariate models to minimize the possibility of spurious results: child age (in years), child sex (male = 1), child race/ethnicity (Black, Hispanic, and Other, with White as the reference category), Household Poverty Ratio as a function of the Federal Poverty Level (FPL) (categories are FPL 100–199%, 200–399%, and 400+%, with <100% as the reference category), neighborhood disorder (a standardized index of the following six items pertaining to social and physical disorder: (1) people in the neighborhood help each other out (reverse coded); (2) people in the neighborhood watch out for other children (reverse coded); (3) when we encounter difficulties, we know where to go for help in our community (reverse coded); (4) in the neighborhood, there is vandalism such as broken windows or graffiti; (5) in the neighborhood, there is poorly kept or rundown housing; and (6) in the neighborhood, there is litter or garbage on the street or sidewalk (alpha = 0.72) [45], maternal age at birth (in years), parent education (1 = less than high school, 2 = high school, 3 = Some college or associate degree, 4 = College degree or higher), parent marital status (married = 1), parent immigrant status (immigrant = 1), and child insurance status (private and public insurance, with uninsured as reference category).

### 2.5. Plan of Analysis

The analysis proceeded as follows. First, descriptive statistics for all variables included in the analyses were calculated. Second, multivariate models including all covariates were estimated. Specifically, multinomial logistic regression was employed to examine the change in the relative risk of a single health condition or multiple health conditions (relative to none) in each of the four health dimensions (i.e., health difficulties, chronic physical conditions, developmental disorders, and mental health conditions) attributable to parental incarceration. Furthermore, negative binomial regression was employed to examine the change in the rate of health conditions in each of the four health dimensions due to parental incarceration. Negative binomial regression was employed in these models given health count outcomes that are zero-inflated and over-dispersed. Third, the association between parental incarceration and health conditions across dimensions was estimated using multinomial logistic regression. The initial modeling strategy took a traditional approach by first comparing those who experienced parental incarceration to those without incarceration, net of covariates. However, a second, more novel strategic comparison approach was then employed which garners estimates pertaining to incarceration but alters the reference category—first to No Incarceration, No Adversity, then to No Incarceration, Single Adversity, and then to No Incarceration, Multiple Adversities. By creating groups that vary in adversity exposure, this approach uses fitting comparisons that can help account for unobserved heterogeneity, and in so doing, “enables a more fine-tuned investigation” of whether parental incarceration is indeed a particularly potent form of adversity/hardship with unique effects on child health [36]. Finally, a figure was constructed that plots the predicted probability of none, one, or two or more health conditions across dimensions by the parent incarceration/child adversity groupings, in an effort to illustrate whether the association between parental incarceration and child health holds when strategic comparison methods are employed.

We conducted all analyses in STATA 15.1 using multiply imputed data (chained equations, 20 imputations). We adjusted all models for cohort-specific fixed effects and included sample weights that adjust for nonresponse, probability of selection, and the demographic distribution of the target population.

## 3. Results

The descriptive results shown in Table 1 reveal that chronic physical conditions are the most common (>26% have one or more), followed by health difficulties (>21% have one or more). Comparatively, nearly 16% have one or more mental health conditions and just over 10% of children have one or more developmental disorders. Ancillary analyses reveal that 53.8% of participants have health conditions in none of the dimensions, 25.9% in only one dimension, 13.9% in two dimensions, 4.8% in three dimensions, and 1.6% in all four dimensions. In terms of parental incarceration, 6.4% of children have experienced the incarceration of a parent. Among the remaining 93.6% of children who did not experience parental incarceration, most experienced no other form of adversity (just under 60% of the full sample). However, a subset experienced other forms of adversity, with 21.4% of the full sample reporting a single adversity (other than incarceration), and 12.6% of the full sample reporting multiple adversities (other than incarceration). Regarding demographics, the average age of children in the sample was approximately 9 years, with just over half (51.6%) of children being male. Most of the children in the sample were white (just under 70%). Most parents had attended college or obtained a college degree, though 11% of children lived in a household below the poverty line. The average maternal age at birth was approximately 30 years, and most parents (72.5%) were married. Finally, 12.3% of children had immigrant parents and most children were insured (72.3% possessed private insurance, while 23.1% possessed public insurance).

Next, we examined the link between parental incarceration and each of the child health dimensions using a more traditional approach. The findings, which are displayed in Table 2, reveal that, overall, parental incarceration is associated with an increased risk of health challenges across all dimensions, particularly when examining the presence of multiple health challenges in each dimension. For instance, parental incarceration is associated with a 57% increase in the relative risk of multiple health difficulties (relative to none), a 26% increase in the relative risk of multiple chronic physical conditions (relative to none), a 58% increase in the relative risk of multiple developmental disorders (relative to none), and a 130% increase in the relative risk of multiple mental health conditions (relative to none). Parental incarceration is also associated with an increased rate of conditions across all four dimensions, ranging from a 12% increase in the rate of chronic physical conditions to a 93% increase in the rate of mental health conditions. Thus, overall, parental incarceration is generally associated with an increased risk of health challenges among children when employing a traditional modeling approach that compares incarceration exposed children to those without incarceration exposure, while controlling for observed covariates.

Although covariates are suppressed in Table 2, these analyses revealed that a number of covariates were robustly and significantly associated with risk in most or all of the health dimensions, including age (i.e., older children manifest more health problems), male (male children manifest more health problems, except in the case of health difficulties), neighborhood disorder (children from neighborhoods characterized by greater disorder exhibit more health challenges in each dimension), parent immigrant status (children with immigrant parents exhibit fewer health challenges in each dimension), and private insurance status (children with private insurance coverage exhibit fewer health challenges in each dimension). Other covariates typically had null or inconsistent associations with the health dimensions. However, non-white children exhibited fewer mental health challenges among this sample, whereas children in households above the poverty line exhibited fewer health difficulties (despite Federal Poverty Level having no association with chronic physical conditions or developmental disorders).

Next, we estimated the relative risk of manifesting health conditions across a varying number of health dimensions (0–4) for incarceration-exposed children. The results are displayed in Table 3. First, we took the traditional approach using all children who had not been exposed to parental incarceration as the reference category, but subsequently we employed strategic comparisons to see if the general pattern of significant findings in Table 2 holds upon employing more comparable adversity-exposed (but not incarceration-exposed) reference groups. The results of the traditional model (Incarceration vs. No Incarceration) indicate that the rate of health conditions across dimensions is 30% higher among incarceration-exposed children (relative to non-exposed children). Furthermore, while incarceration-exposed children incur only a 35% increase in the relative risk of having health conditions in a single dimension (relative to none of the dimensions), they incur a 149% increase in the relative risk of having health conditions across all four dimensions (relative to none of the dimensions).

This pattern of results, however, is altered drastically upon estimating results that use differing strategic comparison groups defined by exposure to other forms of adversity (apart from parental incarceration). For instance, the association between parental incarceration and health conditions across dimensions is particularly robust when estimated with children untouched by adversity as the reference group. To illustrate, compared to children experiencing no adversity, incarceration-exposed children incur a 577% increase in the relative risk of having health conditions across all four dimensions (relative to none of the dimensions). Count models reveal that the rate of health conditions across dimensions is 77% higher among incarceration-exposed children (relative to non-exposed children with no adversity). However, relative to the “no adversity” comparison models, the effects of incarceration are substantially diminished when adversity-exposed children are employed as the reference group, and attenuated to non-significance when children exposed to multiple adversities are employed as the reference group. For instance, compared to children experiencing a single adversity, incarceration-exposed children incur a 307% increase in the relative risk of having health conditions across all four dimensions (relative to none of the dimensions). Count models reveal that the rate of health conditions across dimensions is 35% higher among incarceration-exposed children (relative to non-exposed children with a single adversity). These numbers, however, attenuate to non-significance one children exposed to multiple adversities are employed as the reference group. Specifically, incarceration-exposed children are comparable to children experiencing multiple adversities (but not parent incarceration) in their relative risk of having health conditions across one, two, three, and all four dimensions. In sum, none of the estimates pertaining to incarceration’s impact on health conditions across dimensions attained statistical significance once children exposed to multiple adversities (but not parent incarceration) were employed as the reference group. Count models reveal that the rate of health conditions across dimensions is also not significantly higher among incarceration-exposed children relative to non-exposed children experiencing multiple adversities.

**Figure 1:**
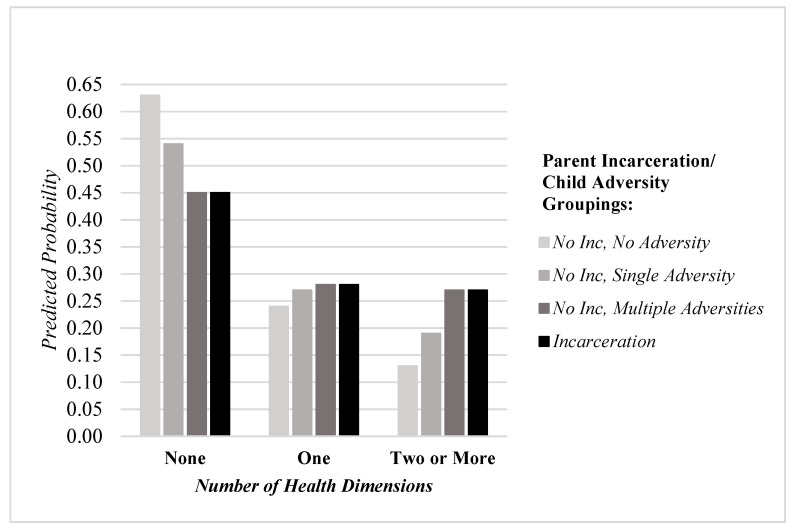
The Predicted Probability of Health Conditions across Dimensions at the Nexus of Incarceration and Child Adversities.

These findings are further illustrated in Figure 1, which plots the predicted probability of none, one, or two or more health conditions across dimensions by the parent incarceration/child adversity groupings. The findings illustrate a pattern in which increasing adversity exposure among the group reporting no parental incarceration corresponds to a predicted probability of health problems across dimensions that essentially matches those of incarceration-exposed children. To illustrate this pattern, while the predicted probability of no health conditions across health dimensions is 0.63 among children exposed to no adversity, it is reduced to 0.45 among children exposed to incarceration. While this suggests a deleterious effect of incarceration on child health, this effect completely disappears when modeled in reference to children exposed to multiple adversities (but not incarceration). For instance, the predicted probability of no health conditions across health dimensions is the same (0.45) among both incarceration-exposed children and children exposed to multiple adversities. When examining the clustering of health problems across health dimensions, the same general principle emerges in the results. For example, while the predicted probability of multiple health conditions across dimensions is only 0.13 among children exposed to no adversity, it increases to 0.27 among children exposed to incarceration. Again, while this suggests a deleterious effect of incarceration on health, this effect completely disappears when modeled in reference to children exposed to multiple adversities (but not incarceration). For instance, the predicted probability of multiple health conditions across dimensions is the same (0.27) among both incarceration-exposed children and children exposed to multiple adversities.

Finally, we conducted a set of ancillary analyses examining the effects of parental incarceration on health when modeled in reference to distinct adversity groupings classified by five themes: family disruption, violence exposure, family mental health, discrimination, and material hardship (for more details, see the Appendix A). The findings indicate that, in the absence of adversity in each of these groupings, incarceration corresponds to significant increases in the rate of health conditions across dimensions, ranging from 32–52%. However, the effects of incarceration become null when the presence of adversity in the following categories is employed as the reference group: violence exposure, family mental health, discrimination, or material hardship. Notwithstanding the general null findings when strategic comparison groups are employed by adversities grouped by theme, the effects of parental incarceration on the rate of health conditions across dimensions is attenuated, but nonetheless significant, when compared to children experiencing family disruption in the form of either death or divorce.

## 4. Discussion

Using nationally representative data from the National Survey of Children’s Health 2016–2018, the aim of this study was to examine the association between parental incarceration and child health across multiple health dimensions. Following this, we employed a strategic comparison modeling approach to assess whether parental incarceration is independently associated with child health outcomes when compared to children who have experienced other forms of childhood adversity (but not parental incarceration). The results of this study can be summarized in three main findings.

First, parental incarceration increases the risk for poorer child health across all four dimensions of health analyzed here (health difficulties, chronic physical conditions, developmental disorders, and mental health conditions) in traditional models that compare incarceration-exposed children to those who have not been exposed to parental incarceration. In many cases, parental incarceration was associated with the experience of multiple health problems within each of the four major dimensions. These results largely confirm the findings of prior research demonstrating that parental incarceration degrades child health outcomes [9,14,15,16,17], while further contributing to the literature by demonstrating this relationship across multiple dimensions of health previously unexplored.

Second, our strategic comparison group models demonstrate the importance of reference group choice when assessing the influence of parental incarceration exposure on child health. Although incarceration exposure corresponds to heightened risk for child health problems when compared to children without incarceration exposure, these effects are substantially attenuated when the reference group is altered to reflect children who have been exposed to other forms of adversity but not parental incarceration. We find the association between parental incarceration and poorer health across health dimensions is attenuated when making comparisons to children facing one alternative (i.e., non-incarceration) form of adversity. Notably, however, the association between parental incarceration and poorer health across health dimensions is fully attenuated when making comparisons to children who experience multiple alternative forms of adversity. This finding implies that exposure to parental incarceration may not necessarily exert an independent influence on child health in the context of a multiplicity of other forms of adversity such as violence exposure, discrimination, family mental health concerns, or material hardship.

Finally, although the results largely demonstrate there is no statistically independent influence of parental incarceration on child health outcomes when compared to those that experience numerous forms of adversity, the experience of family disruption is a notable exception. When compared to children who have experienced a family member’s death or divorce, parental incarceration remains associated with poorer child health outcomes. Parental incarceration may be particularly deleterious for children’s health relative to other forms of family disruption, since the removal of a family member due to their incarceration may be especially destabilizing to the child’s environment. This corroborates research conducted by Geller and colleagues [46,47], which suggests that separation from a parent via incarceration exerts stronger negative effects than other forms of parental separation. One possible explanation is that a forced removal of a parent from a household is a uniquely stigmatizing experience compared to these other forms of adversity. As Johnson and Easterling [31] have previously suggested, “unlike separation due to parental death, divorce, or hospitalization, stigma associated with incarceration may discourage parents and caregivers from discussing the nature of the parent’s absence with children and undermine children’s access to social support.”

### 4.1. Policy Implications

Taken together, the main findings of this study show that incarceration-exposed children are vulnerable to experiencing poorer health outcomes compared to children who have not had a parent incarcerated. Even so, addressing parental incarceration exposure in isolation in an effort to improve child health may not be an effective strategy if the child is embedded in a context of multiple or co-occurring forms of adversity. Families with an incarcerated parent face significant and numerous forms of hardship that are likely to contribute to negative outcomes for children [24]. Thus, reducing exposure to incarceration may not be a broad enough intervention strategy to address larger child health disparities without addressing the array of hardships often experienced by children most at risk for incarceration exposure in the first place [36,41]. Similarly, even in the absence of incarceration, children with multiple adversities experience a similar risk of health problems as children exposed to incarceration, suggesting that incarceration, while important, is only one form of adversity capable of producing health inequities among children. Ultimately, given the relevance of diverse forms of adversity and family stressors for child health, our results point to the need for incarceration-exposed families to have access to holistic and ongoing social services that address multiple domains of hardship such as economic hardship and violence exposure to prevent downstream health consequences among children [24]. For social workers and child advocates, parental incarceration may signal a particularly urgent need for assistance that includes an inventory of the other hardships faced by children within the family [48].

Additionally, incarceration of a parent may serve to further destabilize families already experiencing hardship through events like divorce or the death of a family member, leading to greater risk for poorer health across the dimensions explored here. Resources and programs to assist children in the event of parental incarceration should be especially focused on establishing a stable home environment and assisting in the event of alternative care requirements while paying attention to the family’s history of disruption and its impact on the child [49]. Families with recent disruptions that also face incarceration may benefit particularly from intervention strategies for children through schools to reduce behavioral, academic, and emotional problems that can lead to poorer health [50]. Youth mentoring programs that encourage prosocial attachments and support alternative caregiving relationships by relatives such as grandparents may be especially impact for a child’s well-being within the combined circumstances of parental incarceration and additional family disruption [51,52].

### 4.2. Limitations and Future Research

There are certain limitations for this study that provide opportunities for future research. First, due to the cross-sectional design of the NSCH study, we cannot make claims regarding the causal ordering of the key variables of interest. However, significant prior research suggests that parental incarceration leads to poorer child health outcomes [10,11,12,13,14,15,16], rather than the opposite causal relationship, where child health issues result in subsequent parental incarceration. The use of longitudinal data in future research can help to confirm the causal dynamics of the main research posed here. Second, the measure of incarceration used here does not indicate which parent has been incarcerated. Given research that shows different outcomes for a child’s exposure to maternal versus paternal incarceration [53,54], future studies using a similar comparison group strategy would benefit from the use of a more precise measure of parental incarceration. Third, our analyses were limited to the inclusion of the different types of adversity available in the NSCH dataset such as material hardship, family mental health difficulties, violence exposure, and discrimination. Both researchers and practitioners should consider additional forms of hardship when examining child health disparities as they relate to incarceration exposure.

## 5. Conclusions

The present findings suggest that parental incarceration should be addressed within the context of diverse family stressors and forms of child adversity, and that parental incarceration may not carry unique implications for child health beyond the clustering of other family stressors and child adversities. Policies designed to target parental incarceration as a means of promoting child health could be enhanced to the extent that they adequately address co-occurring family stressors and child adversities.

## Figures and Tables

**Table 1:** Descriptive Statistics.

Variables	Mean	Proportion	SD	Range
**Health Difficulties**				
None	-	0.783	0.412	0–1
Single	-	0.151	0.358	0–1
Multiple	-	0.066	0.250	0–1
Count	0.315	-	0.704	0–7
**Chronic Physical Conditions**				
None	-	0.736	0.441	0–1
Single	-	0.209	0.406	0–1
Multiple	-	0.055	0.229	0–1
Count	0.322	-	0.584	0–6
**Developmental Disorders**				
None	-	0.896	0.305	0–1
Single	-	0.056	0.229	0–1
Multiple	-	0.048	0.214	0–1
Count	0.192	-	0.671	0–6
**Mental Health Conditions**				
None	-	0.842	0.365	0–1
Single	-	0.083	0.276	0–1
Multiple	-	0.075	0.263	0–1
Count	0.309	-	0.808	0–4
**Health Conditions across Dimensions**	0.743	-	0.969	0–4
**Parental Incarceration Permutations**				
No Inc, No Adversity	-	0.596	0.491	0–1
No Inc, Single Adversity	-	0.214	0.410	0–1
No Inc, Multiple Adversities	-	0.126	0.332	0–1
Incarceration	-	0.064	0.245	0–1
**Covariates:**				
Age	9.426	-	5.254	0–17
Male	-	0.516	0.500	0–1
White	-	0.697	0.460	0–1
Black		0.061	0.239	0–1
Hispanic	-	0.113	0.317	0–1
Other Race/Ethnicity	-	0.129	0.335	0–1
FPL < 100%	-	0.110	0.313	0–1
FPL 100–199%	-	0.159	0.366	0–1
FPL 200–399%	-	0.307	0.461	0–1
FPL 400+%	-	0.424	0.494	0–1
Neighborhood Disorder	-	0.000	1.000	−2.995–3.713
Maternal Age at Birth	30.053	-	5.845	18–45
Parent Education	3.441	-	0.801	1–4
Parent Marital Status	-	0.725	0.447	0–1
Parent Immigrant Status	-	0.123	0.328	0–1
Private Insurance	-	0.723	0.447	0–1
Public Insurance	-	0.231	0.422	0–1

**Table 2:** The Link between Parental Incarceration and Child Health: The National Survey of Children’s Health, 2016–2018 (*N* = 102,341).

	**Health Difficulties**		
	**Single**	**Multiple**	**Count**
	**RRR**	**RRR**	**IRR**
	**CI**	**CI**	**CI**
Parental Incarceration	1.07	1.57 **	1.29 **
	0.92–1.24	1.29–1.91	1.15–1.46
	**Chronic Physical Conditions**		
	**Single**	**Multiple**	**Count**
	**RRR**	**RRR**	**IRR**
	**CI**	**CI**	**CI**
Parental Incarceration	1.09	1.26 *	1.12 *
	0.96–1.25	1.01–1.59	1.01–1.24
	**Developmental Disorders**		
	**Single**	**Multiple**	**Count**
	**RRR**	**RRR**	**IRR**
	**CI**	**CI**	**CI**
Parental Incarceration	1.73 **	1.58 **	1.51 **
	1.40–2.14	1.26–1.98	1.30–1.75
	**Mental Health Conditions**		
	**Single**	**Multiple**	**Count**
	**RRR**	**RRR**	**IRR**
	**CI**	**CI**	**CI**
Parental Incarceration	1.83 **	2.30 **	1.93 **
	1.54–2.17	1.95–2.72	1.73–2.15

** *p* < 0.01; * *p* < 0.05. Covariates are included by suppressed to converse space.

**Table 3:** Parental Incarceration and Child Health across Dimensions: A Strategic Comparison Approach.

Number of Dimensions:		Health Conditions across Dimensions					
Parental Incarceration		**None**	**One**	**Two**	**Three**	**Four**	**Count**
**Incarceration v.** **No Incarceration**	RRR/IRR (CI)	Ref	1.34 ** (1.14–1.56)	1.44 ** (1.21–1.70)	2.16 ** (1.73–2.68)	2.49 ** (1.81–3.42)	1.30 ** (1.22–1.38)
Child Adversity		**None**	**One**	**Two**	**Three**	**Four**	**Count**
**Incarceration v.** **No Adversity**	RRR/IRR (CI)	Ref	1.70 ** (1.43–2.02)	2.32 ** (1.92–2.80)	4.82 ** (3.71–6.26)	6.77 ** (4.59–10.01)	1.77 ** (1.64–1.90)
**Incarceration v.** **Single Adversity**	RRR/IRR (CI)	Ref	1.29 ** (1.09–1.52)	1.45 ** (1.22–1.74)	2.58 ** (2.03–3.27)	3.07 ** (2.18–4.33)	1.35 ** (1.26–1.44)
**Incarceration v.** **Multiple Adversities**	RRR/IRR (CI)	Ref	1.03 (0.87–1.24)	0.91 (0.76–1.11)	1.11 (0.88–1.40)	1.20 (0.85–1.70)	1.02 (0.95–1.09)

Note: ** *p* < 0.01. RRR = Relative Risk Ratio; CI = Confidence Interval. Covariates are suppressed to conserve space. All models are weighted to represent the U.S. population of 3- to 5-year-old children and adjust for survey year to account for year-specific fixed effects.

## Data Availability

Data used in the current study are available at: https://www.childhealthdata.org/learn-about-the-nsch/NSCH.

## Appendix A

**Table A1:** Parental Incarceration and Child Health ACROSS Dimensions: Strategic Comparison Approach by Adversity Subcategory.

Form of Child Adversity:		Health Conditions across Dimensions				
		Family Disruption	Violence Exposure	Family Mental Health	Discrimination	Material Hardship
**Incarceration v.** **Absence of Adversity**	IRR (CI)	1.39 ** (1.30–1.49)	1.35 ** (1.27–1.44)	1.42 ** (1.33–1.51)	1.32 ** (1.24–1.40)	1.52 ** (1.42–1.62)
**Incarceration v.** **Presence of Adversity**	IRR (CI)	1.22 ** (1.14–1.30)	0.97 (0.89–1.05)	0.93 (0.87–1.00)	1.03 (0.93–1.14)	1.02 (0.95–1.09)

Note: ** *p* < 0.01; IRR = Incidence Rate Ratio; CI = Confidence Interval. Covariates are suppressed to conserve space. All models are weighted to represent the U.S. population of 3- to 5-year-old children and adjust for survey year to account for year-specific fixed effects. Family disruption includes parent death and/or divorce. Violence exposure includes domestic violence exposure and/or neighborhood violent victimization. Family mental health includes living with someone who has a mental illness or a substance use problem. Discrimination and material hardship are in reference to those specific adversities.
